# Physiological Regulation of Nutritional and Metabolic Biomarkers in Obesity: Implications for Precision Nutrition

**DOI:** 10.3390/nu18061014

**Published:** 2026-03-23

**Authors:** Girolamo Di Maio, Maria Giovanna Tafuri, Maria Casillo, Antonietta Messina, Salvatore Allocca, Ines Villano, Fiorenzo Moscatelli, Antonietta Monda, Marco La Marra, Vincenzo Monda

**Affiliations:** 1 Department of Psychology and Health Sciences, Pegaso Telematic University, 80143 Naples, Italy; 2 Department of Literary, Pegaso University, 80143 Naples, Italy; 3 Section of Human Physiology, Unit of Dietetics and Sports Medicine, Department of Experimental Medicine, University of Campania “Luigi Vanvitelli”, 80138 Naples, Italy; 4 Department of Precision Medicine, University of Campania “Luigi Vanvitelli”, 80138 Naples, Italy; 5 Department of Education and Sport Sciences, Pegaso Telematic University, 80143 Naples, Italy; 6 Department of Human Science and Quality of Life Promotion, San Raffaele Telematic University, 00166 Rome, Italy; 7 Department of Economics, Law, Cybersecurity, and Sports Sciences, University of Naples “Parthenope”, 80131 Naples, Italy

**Keywords:** obesity, biomarkers, precision nutrition, metabolic syndrome, insulin resistance, chronic inflammation, adipokines, lipid metabolism, gut microbiota, personalized medicine

## Abstract

Obesity represents a heterogeneous metabolic disorder characterized by substantial interindividual variation in inflammatory status, insulin sensitivity, and cardiometabolic risk. Traditional anthropometric measures fail to capture this metabolic diversity, limiting risk stratification and personalized intervention strategies. This review critically examines nutritional and metabolic biomarkers that reflect the physiological dysregulation underlying obesity, including adipokines (leptin, adiponectin, resistin), inflammatory markers (*C*-reactive protein, interleukin-6, TNF-α), insulin resistance indices (HOMA-IR, fasting insulin, HbA1c), and lipid metabolism indicators (LDL cholesterol, triglycerides, HDL cholesterol, and liver enzymes such as ALT and GGT). Among these, elevated CRP, reduced adiponectin, and increased HOMA-IR have demonstrated the strongest clinical utility for early metabolic risk identification. We further evaluate emerging biomarkers—including circulating microRNAs, gut microbiota-derived metabolites (short-chain fatty acids, TMAO, lipopolysaccharides), and bile acid profiles—which offer additional mechanistic insight into diet–microbiome–host interactions. We systematically assess the mechanistic basis, clinical relevance, and nutritional modulation of each biomarker class, emphasizing how dietary composition—particularly fatty acid quality, fiber intake, and overall dietary patterns such as the Mediterranean diet—influences biomarker profiles and metabolic outcomes. Furthermore, we explore how biomarker-based phenotyping enables precision nutrition approaches by identifying individuals most likely to benefit from specific dietary interventions. Integration of multi-biomarker panels with clinical and genetic data holds promise for advancing from population-based dietary guidelines toward individualized nutrition strategies that optimize metabolic health and prevent obesity-related complications. Future research should prioritize validating biomarker-guided intervention frameworks, establishing standardized thresholds across diverse populations, and developing clinically implementable tools for personalized nutritional medicine.

## 1. Introduction

Obesity has reached epidemic proportions globally, affecting over one billion individuals worldwide and representing one of the leading drivers of preventable morbidity and mortality across all age groups and income settings [1]. Its rising prevalence is paralleled by an escalating burden of associated comorbidities, including type 2 diabetes mellitus, cardiovascular disease, non-alcoholic fatty liver disease, and certain malignancies, imposing substantial costs on both healthcare systems and individual quality of life [2]. Obesity is traditionally defined by excess body fat accumulation and classified using simple anthropometric indices such as body mass index (BMI). However, growing physiological and clinical evidence demonstrates that obesity is not a uniform condition but rather a heterogeneous metabolic disease characterized by marked interindividual variability in metabolic function, inflammatory status, hormonal regulation, and risk of cardiometabolic complications [3]. Individuals with similar BMI values may display profoundly different metabolic phenotypes, ranging from relatively preserved insulin sensitivity to severe metabolic dysfunction, highlighting the complexity of obesity beyond excess adiposity alone [4].

The widespread use of anthropometric measures has undeniable advantages in terms of feasibility and population-level surveillance, yet these indices provide limited insight into the underlying metabolic and physiological alterations associated with obesity [5]. BMI and waist circumference do not capture differences in adipose tissue distribution, adipocyte function, ectopic lipid accumulation, inflammatory activation, or metabolic flexibility. As a result, reliance on anthropometry alone may obscure early pathophysiological changes, delay risk stratification, and limit the ability to monitor individual responses to nutritional and lifestyle interventions [6].

In this context, nutritional and metabolic biomarkers have emerged as valuable tools to characterize the physiological and pathophysiological processes underlying obesity. Biomarkers reflecting adipose tissue endocrine function, low-grade inflammation, insulin resistance, lipid metabolism, micronutrient status, and gut microbiota-derived metabolites provide mechanistic insights into energy homeostasis and metabolic regulation [7]. Importantly, these biomarkers are not merely descriptive but reflect dynamic processes influenced by dietary composition, nutrient availability, and metabolic adaptability, thereby linking nutritional exposures to physiological outcomes [8].

The integration of biomarker-based phenotyping into obesity research has important implications for precision medicine and precision nutrition. By capturing individual variability in metabolic responses and regulatory pathways, biomarkers may support more refined classification of obesity phenotypes, improve prediction of disease risk, and guide personalized nutritional strategies aimed at restoring metabolic homeostasis [7].

The aim of this review is therefore to critically examine nutritional and metabolic biomarkers in obesity from a physiological perspective, highlighting their mechanistic basis, clinical relevance, and potential role in informing precision nutrition approaches. By integrating classical and emerging biomarkers within a framework of metabolic physiology, this review seeks to contribute to a more nuanced understanding of obesity and to support the development of individualized, physiology-guided interventions.

For the purposes of this review, “nutritional biomarkers” are defined as measurable biological molecules or indices whose circulating or tissue concentrations are directly modulated by dietary intake, nutritional status, or specific food components and which reflect physiological processes relevant to energy metabolism, inflammation, or cardiometabolic risk [9]. This category encompasses classical micronutrient markers (e.g., 25-hydroxyvitamin D, serum ferritin, plasma B12) and dynamic metabolic indices whose levels respond quantifiably to dietary interventions, including adipokines, inflammatory cytokines, insulin resistance indices, lipid fractions, miRNAs, and gut microbiota-derived metabolites. By defining this scope explicitly, the review distinguishes nutritional biomarkers from purely anthropometric measures and from static genetic or epigenetic markers that do not reflect acute or chronic dietary exposures.

This narrative review was conducted following a systematic literature search of PubMed/MEDLINE, Scopus, and Web of Science for articles published between January 2000 and February 2026. Search terms included combinations of “obesity”, “biomarkers”, “precision nutrition”, “metabolic syndrome”, “adipose tissue”, “insulin resistance”, “inflammation”, “lipid metabolism”, “microRNA”, “gut microbiota”, “micronutrients”, and “personalised medicine”. Inclusion criteria required original research articles, systematic reviews, and meta-analyses published in English addressing mechanistic, clinical, or nutritional aspects of the biomarkers discussed. References were further enriched by manual screening of retrieved article bibliographies, prioritizing methodological rigor, clinical relevance, and recency of evidence.

## 2. Inflammatory and Adipose Tissue-Derived Biomarkers

Adipose tissue is a central regulator of systemic energy homeostasis and endocrine signaling, functioning not only as the primary site for triacylglycerol (TAG) storage but also as a major source of circulating metabolic and inflammatory biomarkers. Through the coordinated regulation of lipid storage and mobilization, adipose tissue actively communicates with peripheral organs via the secretion of adipokines, cytokines, and acute-phase proteins that reflect its functional and inflammatory state [8]. These processes are intimately linked to the regulation of circulating lipoproteins, including low-density lipoprotein (LDL), high-density lipoprotein (HDL), triglycerides (TG), and apolipoprotein B (ApoB), which collectively serve as critical indicators of cardiometabolic risk (Table 1).

Adipocytes, the predominant cell type within adipose tissue, are specialized for lipid storage in large, unilocular lipid droplets. Lipid accumulation occurs through lipogenesis. These anabolic processes are tightly linked to adipokine secretion, particularly adiponectin, which enhances insulin sensitivity and promotes efficient lipid handling. In contrast, lipolysis enables the release of free fatty acids and glycerol during periods of increased energy demand [10]. This process is primarily stimulated by sympathetic nervous system activation through β-adrenergic receptors and protein kinase A (PKA) [11]. Unrestrained lipolysis, as occurs in dysfunctional adipose tissue, floods the liver with non-esterified fatty acids, driving hepatic VLDL-TG and ApoB secretion and generating a pro-atherogenic lipid profile characterized by elevated LDL and TG, increased small dense LDL particles, and reduced HDL cholesterol [12]. Adiponectin facilitates the metabolic utilization of lipolysis-derived fatty acids by activating AMP-activated protein kinase (AMPK) and peroxisome proliferator-activated receptor-α (PPARα) in skeletal muscle and liver, thereby limiting lipid accumulation and insulin resistance [13]. Reduced adiponectin, a hallmark of adipose dysfunction, is associated with impaired TG clearance, elevated ApoB-containing lipoprotein particles, and lower HDL, reinforcing the mechanistic link between adipokine dysregulation and atherogenic dyslipidemia [14].

Beyond their metabolic role, adipocytes secrete a wide array of adipokines, including leptin, adiponectin, and resistin, which serve as key biomarkers of adipose tissue function. Leptin reflects energy stores and regulates appetite and energy expenditure through central nervous system pathways, while adiponectin exerts insulin-sensitizing, anti-inflammatory, and cardioprotective effects, in part by promoting reverse cholesterol transport and supporting HDL functionality [15]. In contrast, resistin has been implicated in the promotion of insulin resistance and metabolic inflammation and has been shown to upregulate hepatic LDL receptor degradation and stimulate ApoB-containing lipoprotein secretion, particularly in the context of obesity [16].

Adipose tissue is also a heterogeneous organ containing a substantial stromal-vascular fraction (SVF), composed of preadipocytes, immune cells, endothelial cells, and progenitor cells [17]. Dysregulation of adipose tissue expansion is accompanied by immune cell infiltration and increased production of pro-inflammatory cytokines, including tumor necrosis factor-α (TNF-α), interleukin-6 (IL-6), and interleukin-1β (IL-1β) [18,19]. Systemic inflammation associated with adipose tissue dysfunction is further reflected by elevated levels of acute-phase proteins, particularly *C*-reactive protein (CRP), which is largely induced by IL-6 signaling in the liver. Circulating CRP levels are widely used as a clinical biomarker of obesity-associated inflammation and cardiometabolic risk [20].

Collectively, alterations in adipokine profiles, increased pro-inflammatory cytokine production, and elevated acute-phase proteins represent a hallmark of dysfunctional adipose tissue. Reduced adiponectin levels, together with increased leptin, resistin, TNF-α, IL-6, IL-1β, and CRP, link adipose tissue inflammation to insulin resistance, type 2 diabetes, cardiovascular disease, and other obesity-related metabolic disorders [21]. These perturbations are mirrored in the circulating lipoprotein profile, where elevated TG, LDL, and ApoB, alongside reduced HDL, constitute an atherogenic signature that amplifies cardiovascular risk beyond that predicted by any single biomarker alone. ApoB deserves particular emphasis in this context, as it enumerates the total number of circulating atherogenic lipoprotein particles—encompassing VLDL, intermediate-density lipoprotein (IDL), and LDL—and may therefore capture residual cardiometabolic risk not fully reflected by LDL cholesterol concentrations [22].

In addition to intrinsic adipose tissue dysfunction, dietary factors critically modulate adipokine secretion, inflammatory biomarker profiles, and lipoprotein metabolism. The quality of dietary fat plays a pivotal role, with monounsaturated and omega-3 polyunsaturated fatty acids (MUFAs and *n*−3 PUFAs), typical of plant oils, nuts, and fish, promoting adipocyte insulin sensitivity and favoring an anti-inflammatory adipokine pattern characterized by increased adiponectin and reduced TNF-α and IL-6 levels [23]. MUFAs have additionally been associated with reductions in LDL and ApoB without adverse effects on HDL, while *n*−3 PUFAs are particularly effective in lowering fasting TG through suppression of hepatic VLDL synthesis [24]. In contrast, diets enriched in saturated and trans fatty acids exacerbate adipose tissue inflammation, macrophage infiltration, and the secretion of pro-inflammatory cytokines, contributing to insulin resistance [25]. These dietary fats also upregulate LDL and ApoB concentrations, suppress HDL, and impair TG clearance, further worsening the atherogenic lipid profile [26]. Dietary fiber further influences adipose tissue biology by improving glycemic control and promoting gut microbiota-derived short-chain fatty acids, which enhance adiponectin expression and suppress inflammatory signaling [27], and has independently been shown to reduce LDL and TG levels while supporting HDL metabolism. Likewise, dietary antioxidants, including polyphenols and vitamins with redox activity, attenuate oxidative stress within adipose tissue, thereby limiting cytokine production and preserving insulin sensitivity [28], with certain polyphenols also demonstrating favorable effects on LDL oxidation and HDL functionality [26].

These nutritional effects are reflected at the dietary-pattern level. The Mediterranean diet, characterized by high intakes of MUFAs, fiber, and antioxidant-rich plant foods, is consistently associated with a favorable biomarker profile, including higher circulating adiponectin and lower levels of leptin, resistin, TNF-α, IL-6, IL-1β, and CRP [29]. At the lipoprotein level, this dietary pattern is associated with reduced LDL, lower TG, decreased ApoB, and preserved or elevated HDL, supporting its established role in cardiovascular risk reduction [30]. Conversely, the Western diet, rich in saturated fats, refined carbohydrates, and ultra-processed foods, promotes adipose tissue dysfunction and chronic low-grade inflammation, as evidenced by reduced adiponectin and elevated pro-inflammatory cytokines and CRP [31], and is correspondingly linked to an atherogenic lipoprotein pattern of high TG, high LDL, elevated ApoB, and low HDL. Collectively, these findings highlight diet quality as a key modulator linking adipose tissue biology to systemic metabolic and cardiometabolic risk, with HDL, LDL, TG, and ApoB serving as integrative biomarkers that capture the downstream lipoprotein consequences of adipose tissue health and dietary exposures.

## 3. Metabolic and Insulin Resistance Biomarkers

Insulin resistance is defined by a reduced responsiveness of target tissues to insulin, leading to impaired glucose uptake, disrupted lipid metabolism, and progressive metabolic dysfunction. This condition represents a pivotal pathophysiological link between obesity and the development of type 2 diabetes (T2D). The identification of robust and reliable biomarkers is therefore essential not only for diagnosis and risk stratification but also for predicting the transition from obesity to T2D and for monitoring metabolic responses to dietary or weight-loss interventions [32].

Currently used biomarkers—including HOMA-IR, fasting insulin levels, and HbA1c—provide complementary information on insulin sensitivity, pancreatic compensation, and long-term glycemic exposure [33].

Beyond their diagnostic utility, longitudinal changes in these markers enable the identification of individuals with obesity who are most likely to progress toward T2D and allow clinicians to evaluate the effectiveness of lifestyle or pharmacological interventions aimed at improving insulin sensitivity. As research continues to refine the biomarker landscape, integrating classical metabolic indices with inflammatory and molecular markers is becoming increasingly relevant for personalized prevention strategies.

### 3.1. HOMA-IR as a Biomarker

HOMA-IR is a widely used surrogate index for estimating insulin resistance based on fasting glucose and insulin concentrations. Despite its simplicity and cost-effectiveness, considerable variability exists in the threshold values used to define insulin resistance across populations [34]. Population-based studies have proposed cut-off values ranging from approximately 2.3 to 2.5, with differences influenced by age, BMI, ethnicity, and metabolic status [35,36]. These findings underscore the importance of context-specific thresholds when using HOMA-IR to identify insulin resistance and metabolic risk [37]. Importantly, elevated HOMA-IR values in individuals with obesity often precede overt hyperglycemia, making this index particularly valuable for predicting the progression from obesity to T2D [38].

Longitudinal studies demonstrate that persistently high or rising HOMA-IR is associated with increased risk of metabolic syndrome, T2D, and cardiovascular disease. Moreover, reductions in HOMA-IR following caloric restriction, dietary modification, or weight loss provide a practical means of monitoring improvements in insulin sensitivity in response to lifestyle interventions [39]. Although emerging biomarkers such as circulating microRNAs may offer enhanced sensitivity [38]. HOMA-IR remains a cornerstone of both clinical and epidemiological research due to its accessibility and interpretability.

### 3.2. Fasting Insulin Levels

Fasting insulin concentration reflects pancreatic β-cell activity and compensatory hyperinsulinemia in response to insulin resistance. Elevated fasting insulin levels are commonly observed in obesity and represent an early metabolic alteration preceding changes in fasting glucose or HbA1c. As such, fasting insulin is particularly informative for detecting subclinical insulin resistance and identifying individuals at high risk for progression to T2D. Clinical and experimental studies show that increases in fasting insulin correlate with worsening insulin resistance, systemic inflammation, and adipose tissue dysfunction [40,41]. Conversely, declines in fasting insulin following dietary interventions, increased physical activity, or weight loss are indicative of improved insulin sensitivity, even in the absence of major changes in glycemia [42]. This makes fasting insulin a useful biomarker for tracking early metabolic benefits of lifestyle interventions, especially in individuals with obesity or prediabetes.

Additionally, fasting insulin levels interact closely with inflammatory biomarkers such as CRP, TNF-α, and IL-6, reinforcing the concept that hyperinsulinemia and inflammation act synergistically to accelerate metabolic deterioration [43].

### 3.3. HbA1c as a Biomarker

HbA1c remains a central biomarker for the diagnosis and long-term monitoring of glycemic control in T2D. Formed through non-enzymatic glycation of hemoglobin, HbA1c reflects average blood glucose levels over approximately 2–3 months [44]. According to ADA criteria, HbA1c values ≥ 6.5% indicate diabetes, while levels between 5.7% and 6.4% identify individuals with prediabetes [45]. Although HbA1c is less sensitive than HOMA-IR or fasting insulin in detecting early insulin resistance, it plays a critical role in confirming metabolic progression from insulin resistance to overt dysglycemia. Rising HbA1c levels over time signal failure of compensatory mechanisms and β-cell dysfunction, marking the transition toward T2D [46]. From an interventional perspective, HbA1c is a robust marker for monitoring the long-term effectiveness of dietary changes, weight loss, and pharmacological therapies, particularly in individuals with established dysglycemia. However, its interpretation must consider confounding factors such as altered erythrocyte turnover, anemia, liver disease, and hemoglobin variants [47,48,49,50,51,52].

For this reason, HbA1c is most informative when evaluated alongside insulin resistance indices and inflammatory markers.

## 4. Biomarkers of Lipid Dysregulation and Dietary Modulation

Lipid imbalance, marked by atypical lipid levels in the bloodstream, poses a major risk for various diseases, such as cardiovascular disease, metabolic syndrome, obesity, and T2DM. Tracking lipid biomarkers is crucial for the early identification and management of lipid imbalance and cardiovascular disorders. Understanding the fundamental processes of lipid metabolism and recognizing high-risk individuals allows clinicians to implement preventive strategies and initiate appropriate treatment plans, including lifestyle modification, dietary interventions, pharmacological therapy, or a combination of these approaches [53,54].

Importantly, dietary composition and energy intake strongly influence lipid biomarkers, modulating disease risk and progression. A variety of biomarkers are used to assess lipid dysregulation, providing valuable insights into the underlying pathophysiology.

### 4.1. Total Cholesterol as a Biomarker and Dietary Modulation

Total cholesterol represents the overall concentration of cholesterol in the bloodstream, encompassing LDL, HDL, and triglyceride-rich lipoproteins. Elevated total cholesterol levels, particularly increased LDL and reduced HDL concentrations, are well-established risk factors for coronary heart disease [55]. Excess cholesterol also contributes to hepatic lipid accumulation, exacerbating liver inflammation and fibrosis and promoting the progression of MASLD (metabolic dysfunction-associated steatotic liver disease) to MASH [56]. In individuals with T2DM, insulin resistance profoundly alters lipid metabolism, leading to dyslipidemia characterized by increased total cholesterol and triglycerides. This condition markedly elevates the risk of atherosclerosis, highlighting the importance of cholesterol management in diabetes care [57]. Dietary modulation plays a central role in improving cholesterol profiles, often representing the first-line intervention before pharmacological therapy.

### 4.2. Saturated vs. Unsaturated Fats

High intake of saturated fatty acids (SFAs), typically derived from animal fats and ultra-processed foods, is associated with increased LDL cholesterol and enhanced hepatic cholesterol synthesis [58]. In contrast, replacing SFAs with mono- and polyunsaturated fatty acids (MUFAs and PUFAs), particularly from olive oil, nuts, seeds, and fatty fish, has been shown to reduce total and LDL cholesterol while preserving or increasing HDL levels [59]. This dietary substitution improves lipid particle size and reduces atherogenic risk, with additional benefits for insulin sensitivity and hepatic fat accumulation. Atherogenic dyslipidemia—characterized by elevated small dense LDL particles and reduced HDL—is not consistently observed in MASLD alone but becomes an independent risk factor for liver fibrosis when MASLD coexists with T2DM [60]. Dietary patterns rich in unsaturated fats appear particularly effective in mitigating this combined metabolic burden. LDL cholesterol, commonly referred to as “bad” cholesterol, plays a key role in atherosclerotic plaque formation, and elevated levels are consistently linked to cardiovascular mortality and T2DM in population-based studies [60]. Conversely, HDL cholesterol facilitates reverse cholesterol transport from peripheral tissues, including the arterial wall, to the liver and exerts anti-inflammatory, antioxidant, and antithrombotic effects, partly mediated by apoA1 and associated enzymes [61,62]. Low HDL levels have also been associated with advanced liver fibrosis in individuals with T2DM [62]. Alterations in cholesterol metabolism in T2DM include reduced intestinal cholesterol absorption, reflected by lower plasma campesterol levels, and increased endogenous cholesterol synthesis, indicated by elevated lathosterol concentrations. These changes correlate with increased liver fat content and may be driven by enhanced SREBP2 activity, a key regulator of cholesterol uptake and synthesis [63]. Dietary cholesterol restriction and increased intake of plant sterols may partially counteract these metabolic alterations, further supporting dietary intervention as a modifiable determinant of lipid dysregulation.

### 4.3. Triglycerides as a Biomarker and Dietary Modulation

Triglycerides are the primary form of fat storage in the body and serve as an important energy source. However, chronically elevated triglyceride levels are associated with increased risk of cardiovascular disease, stroke, and T2DM [62]. Prolonged exposure to excess lipids, as observed in obesity and metabolic syndrome, promotes triglyceride accumulation in pancreatic β-cells, inducing lipotoxicity. This process triggers inflammatory signaling, β-cell dysfunction, and apoptosis, ultimately contributing to hyperglycemia and hepatic injury, thereby facilitating progression from steatosis to MASH [64]. In T2DM, hypertriglyceridemia arises mainly from increased hepatic production of triglyceride-rich VLDL1 particles and impaired clearance of triglyceride-rich lipoproteins (TRLs). These disturbances are driven by enhanced de novo lipogenesis, reduced insulin-mediated suppression of VLDL secretion, diminished lipoprotein lipase activity, and decreased hepatic uptake of TRL remnants [65].

### 4.4. Omega-3 Fatty Acids

Omega-3 polyunsaturated fatty acids (PUFAs), particularly EPA and DHA, have demonstrated triglyceride-lowering effects through suppression of hepatic lipogenesis, enhanced fatty acid oxidation, and reduced VLDL secretion [59]. Supplementation with omega-3 fatty acids has been associated with improvements in plasma triglyceride levels and may exert anti-inflammatory effects relevant to both cardiovascular disease and MASH [66]. These properties make omega-3 fatty acids a valuable dietary adjunct in individuals with hypertriglyceridemia and metabolic dysfunction.

### 4.5. Caloric Restriction vs. Macronutrient Manipulation

Caloric restriction consistently reduces triglyceride levels by decreasing hepatic fat content and improving insulin sensitivity [67]. However, emerging evidence suggests that macronutrient composition may be as important as total caloric intake. Diets low in refined carbohydrates and added sugars reduce hepatic de novo lipogenesis more effectively than low-fat diets, resulting in greater reductions in triglyceride levels [68]. Thus, targeted macronutrient manipulation, particularly carbohydrate quality and quantity, may provide additional metabolic benefits beyond energy restriction alone.

### 4.6. ALT and AST as Biomarkers in the Context of Diet

Alanine aminotransferase (ALT) and aspartate aminotransferase (AST) are widely used biomarkers for detecting liver injury and are commonly employed in screening individuals with T2DM for advanced fibrosis and MASH [69]. Normal serum ranges are typically 7–55 U/L for ALT and 8–49 U/L for AST. The AST:ALT ratio is usually less than one; ratios greater than two strongly suggest alcoholic liver disease, whereas values below one are more indicative of MASLD or MASH [70] (European Association for the Study of the Liver (EASL) et al., n.d.). Despite their clinical utility, ALT and AST lack sensitivity and specificity for early-stage disease and for distinguishing MASLD from MASH [71]. Diet-induced weight loss and improvements in dietary fat quality have been shown to reduce ALT and AST levels, reflecting decreased hepatic inflammation and lipid accumulation. Nevertheless, these enzymes remain imperfect indicators of disease severity, underscoring the need for complementary biomarkers.

## 5. miRNAs as Biomarkers and Nutritional Regulation

MicroRNAs (miRNAs) are small non-coding RNAs that regulate gene expression at the post-transcriptional level and have emerged as promising biomarkers for metabolic diseases, including T2DM and MASH [72]. Circulating miRNAs, transported in extracellular vesicles, enable intercellular communication and reflect epigenetic regulation without altering DNA sequences. Consequently, plasma miRNA profiles provide insight into an individual’s metabolic and inflammatory state [73] (Table 2).

Several miRNAs have demonstrated clinical relevance specific to obesity. miR-15a and miR-365 are upregulated in adipose tissue of obese individuals and have been associated with impaired adipogenesis and dysregulated lipid storage [74]. miR-375 and miR-222 show altered circulating levels in obesity and are linked to pancreatic β-cell dysfunction and insulin secretion impairment, respectively [75]. These markers have been proposed as early indicators of metabolic deterioration preceding overt T2DM.

A distinct set of miRNAs is shared across MASH and T2DM, reflecting common pathophysiological pathways [38,76,77,78]. Among these, miR-122 is the most extensively validated hepatic biomarker: it is highly enriched in hepatocytes, and reduced circulating levels correlate with hepatic steatosis severity, insulin resistance, and increased cardiovascular risk, with diagnostic performance comparable to conventional liver enzymes in several cohort studies [79]. miR-126, predominantly expressed in endothelial cells, is downregulated in both T2DM and atherosclerosis, and its reduction has been prospectively associated with incident cardiovascular events and impaired vascular insulin signaling [80]. miR-29a regulates extracellular matrix remodeling through collagen gene suppression; its dysregulation has been clinically linked to hepatic fibrosis progression in MASH and to impaired insulin receptor substrate signaling in skeletal muscle [81]. miR-144 modulates glucose transporter expression, and its circulating levels are inversely correlated with fasting glucose and HbA1c in patients with T2DM [82]. miR-103 and miR-223 have been associated with systemic insulin resistance and macrophage-driven hepatic inflammation, respectively, with miR-103 showing particular promise as a marker of dietary fat-induced metabolic stress [83].

Importantly, dietary interventions may influence miRNA expression, suggesting a mechanistic link between nutrition and epigenetic regulation [84]. Diets rich in unsaturated fats, omega-3 fatty acids, and low-glycemic-load carbohydrates have been shown to upregulate miR-126 and downregulate miR-103, modulating pathways involved in lipid metabolism, vascular inflammation, and insulin signaling [84]. These findings support the concept that miRNAs serve not only as diagnostic biomarkers but also as dynamic indicators of dietary responsiveness, with potential utility for monitoring the efficacy of precision nutrition interventions in metabolic disease.

## 6. Gut Microbiota, Biomarkers, and Nutritional Modulation

The gut microbiota refers to the complex community of microorganisms inhabiting the intestinal tract, responsible for producing bioactive metabolites, maintaining metabolic homeostasis, and regulating immune responses. Beyond local gastrointestinal functions, it is connected to the central nervous system through the gut–brain axis, influencing hunger–satiety signaling, dopamine reward pathways, and energy acquisition [85]. This axis is dynamically shaped by genetics, psychological stress, and dietary patterns. Consequently, the gut microbiota is now recognized as a major determinant of interindividual variability in dietary responses, and its direct assessment represents an advanced strategy within precision nutrition frameworks [86].

### 6.1. Microbiota-Derived Biomarkers

Several microbial metabolites have emerged as key biomarkers linking diet, gut microbiota, and metabolic health (Table 3). Short-chain fatty acids (SCFAs)—acetate, propionate, and butyrate—produced through fermentation of dietary fiber, maintain intestinal barrier integrity, regulate immune responses, and modulate glucose and lipid metabolism [87]. Butyrate serves as the primary energy source for colonocytes and exerts anti-inflammatory effects, while propionate and acetate influence hepatic gluconeogenesis and appetite regulation [88]. Trimethylamine *N*-oxide (TMAO), derived from choline and L-carnitine in red meat, is associated with increased cardiovascular risk and insulin resistance [89]. Circulating lipopolysaccharides (LPS) reflect intestinal permeability and microbial translocation, promoting systemic inflammation, insulin resistance, and hepatic steatosis [90]. Microbial-derived bile acids regulate lipid absorption and glucose metabolism via FXR and TGR5 receptors, and their dysregulation contributes to metabolic dysfunction [91].

### 6.2. Dietary Patterns and Microbiota Modulation

Diet is among the most powerful modulators of gut microbiota composition. Animal-based diets promote bile-tolerant microbial species, while plant-based diets favor fiber-degrading bacteria [92]. Even short-term changes—such as a 24 h reduction in carbohydrate intake—can significantly alter microbial populations [93]. Long-term consumption of energy-dense, nutrient-poor diets reduces microbial diversity, particularly with prolonged weight gain [94]. Food additives also play a role: prolonged sucralose intake has been linked to reductions in beneficial bacteria and increased pro-inflammatory gene expression, while excessive emulsifier consumption impairs barrier function and microbial diversity [95]. FODMAP-rich diets may reduce gastrointestinal symptoms and lower insulin resistance risk in overweight individuals, though their long-term effects on microbial diversity warrant careful consideration [96,97,98].

### 6.3. Gluten-Free Diets and Microbial Adaptation

Short-term gluten-free diets (GFDs) produce significant changes in microbiota composition [99]. In healthy individuals, GFDs are associated with decreased levels of beneficial bacteria—including *Bifidobacterium*, *Faecalibacterium prausnitzii*, and *Lactobacillus*—and increased *Enterobacteriaceae* [100]. Low-gluten diets may reduce intestinal gas and improve bloating, but long-term adherence without a medical indication may increase cardiovascular risk through reduced whole-grain and fiber intake [101].

### 6.4. Dysbiosis as a Mediator Between Diet and Metabolic Inflammation

Diet-induced dysbiosis promotes impaired SCFA production, increased endotoxemia, altered bile acid signaling, and elevated TMAO, collectively driving insulin resistance, hepatic fat accumulation, and systemic inflammation [87]. Conversely, diets rich in fiber, polyphenols, and fermented foods support microbial diversity, enhance SCFA production, and reduce inflammatory signaling [102]. This evidence positions the gut microbiome as a key biological interface through which nutrition exerts its metabolic effects, supporting its integration into precision nutrition strategies.

### 6.5. Micronutrient Disturbances in Obesity

Individuals with obesity are at increased risk of deficiencies in essential minerals—particularly zinc, copper, iron, calcium, and magnesium—and in both fat-soluble and water-soluble vitamins [103]. These deficiencies arise from four main mechanisms: dietary inadequacy, increased physiological requirements, altered pharmacokinetics, and impaired absorption. Chronic low-grade inflammation elevates micronutrient demand, while gut dysbiosis, bariatric surgery, and elevated circulating lipids further compromise absorption and distribution [104].

### 6.6. Dietary Deficiency

Micronutrient deficiencies in obesity are frequently linked to poor dietary quality. NHANES data (2001–2008) demonstrate a high prevalence of insufficient micronutrient intake across weight categories, with the most concerning patterns in individuals with overweight and obesity [105]. Ultra-processed foods (UPFs)—low in fiber, micronutrients, and phytochemicals but rich in refined carbohydrates and added sugars—drive both excess energy intake and widespread micronutrient inadequacy and are strongly associated with insulin resistance and T2DM [106,107]. Notably, very-low-calorie diets (VLCDs) prescribed for rapid weight loss can also worsen deficiencies, highlighting the contribution of non-dietary mechanisms beyond intake alone [108].

### 6.7. Implications for Supplementation Strategies

Standard dietary recommendations and low-dose multivitamins may be insufficient when increased requirements, altered pharmacokinetics, and impaired absorption coexist. Adipose tissue sequestration reduces bioavailability of fat-soluble vitamins, while altered lipid profiles can disrupt transport of protein-bound micronutrients [109]. In individuals on VLCDs or post-bariatric surgery, supplementation is critical given further compromised absorption [110,111]. These considerations support personalized, condition-specific supplementation guided by biochemical assessment rather than dietary intake alone, potentially requiring higher doses, specific combinations, or alternative delivery routes. Supplementation should complement—not replace—strategies aimed at improving overall diet quality and reducing UPF consumption.

## 7. Discussion

The evidence reviewed in this paper collectively supports a fundamental reframing of obesity management: from a weight-centric paradigm toward a biomarker-guided, phenotype-specific approach. Biomarkers of adipose tissue function, systemic inflammation, insulin resistance, lipid metabolism, hepatic integrity, and nutritional status do not merely describe metabolic dysfunction—they define it, stratify its severity, and, critically, predict its responsiveness to intervention. Realizing this clinical potential, however, demands a shift from descriptive biomarker science toward actionable, validated, integrated frameworks.

A critical appraisal of the biomarker evidence reviewed here reveals three important unresolved controversies that clinicians and researchers must acknowledge alongside its clinical promise. First, HOMA-IR lacks universally validated cut-off values: population-based studies propose thresholds ranging from 2.0 to 2.9 depending on ethnicity, age, and BMI, reflecting genuine biological differences in insulin secretion between ethnic groups rather than statistical artifacts [112]. Applying a single HOMA-IR threshold across diverse populations risks systematic misclassification and undermines the precision that biomarker-guided nutrition aims to deliver [113]. Second, TMAO has attracted considerable attention as a diet–microbiota–cardiovascular risk biomarker, but the evidence base remains contested: several prospective cohort studies report associations with major adverse cardiovascular events, yet Mendelian randomization analyses and interventional data have produced inconsistent results, and TMAO levels are strongly confounded by renal function, dietary protein intake, and gut microbiota composition [89]. TMAO should therefore be interpreted as a hypothesis-generating rather than an established clinical risk marker pending further validation. Third, the metabolically healthy obesity (MHO) phenotype is demonstrably unstable over time: longitudinal analyses consistently show that 30–50% of individuals initially classified as MHO transition to metabolically unhealthy obesity over a 5–10-year horizon, particularly in the absence of sustained lifestyle modification [114]. This instability has a critical practical implication: serial biomarker monitoring, tracking trajectories of adiponectin, inflammatory markers, lipid fractions, and glycemic indices over time, is essential to detect MHO-to-MUO transitions early and trigger proactive intervention before complications become irreversible.

### 7.1. Biomarkers for Early Diagnosis

Standard diagnostic criteria for metabolic syndrome and type 2 diabetes are, by design, late-stage instruments. They identify individuals after pathophysiological processes—adipose tissue inflammation, ectopic lipid deposition, and progressive beta-cell dysfunction—are already well entrenched [115]. In contrast, biomarker-based approaches may enable earlier identification of at-risk phenotypes by capturing subclinical alterations in insulin sensitivity, lipid metabolism, inflammatory status, and adipose tissue function [113]. The case for early biomarker detection is compelling. Reduced circulating adiponectin and elevated leptin-to-adiponectin ratios precede impaired fasting glucose by years [116], representing a window of opportunity that conventional metrics miss entirely. Similarly, increased levels of pro-inflammatory cytokines such as IL-6 and TNF-α, together with elevated high-sensitivity *C*-reactive protein (hs-CRP), may reflect chronic low-grade inflammation before the manifestation of cardiovascular events or metabolic disease [117]. Lipid-derived biomarkers, including elevated triglycerides, reduced HDL cholesterol, and increased small dense LDL particles, provide additional insights into atherogenic dyslipidemia and cardiovascular risk at early stages [118]. Crucially, each of these biomarkers captures a distinct pathophysiological axis; their combination into composite risk algorithms substantially outperforms any single marker in predictive accuracy.

What is needed now is not more cross-sectional biomarker associations but the development and external validation of composite diagnostic algorithms with clinically meaningful sensitivity and specificity thresholds. Establishing standardized, population-stratified reference ranges—accounting for age, sex, ethnicity, and body composition—is an urgent scientific and regulatory priority.

### 7.2. Phenotyping “Metabolically Healthy vs. Unhealthy Obesity”

The metabolically healthy obesity (MHO) phenotype represents one of the most instructive paradoxes in metabolic medicine: comparable adiposity can coexist with vastly different cardiometabolic risk profiles (Table 4). This heterogeneity is not noise—it is biologically meaningful, and biomarker profiling is the principal tool for resolving it.

Individuals with MHO characteristically display higher adiponectin, lower leptin-to-adiponectin ratios, attenuated pro-inflammatory cytokine secretion (TNF-α, IL-6, IL-1β), lower hs-CRP, more favorable lipid profiles (higher HDL, lower TG, lower ApoB), and better-preserved insulin sensitivity as reflected by HOMA-IR and liver transaminases [114]. This profile is not simply less bad; it reflects fundamentally different adipose tissue biology, with preserved expandability, lower macrophage infiltration, and maintained adipokine signaling.

What the literature has also made clear, however, is that MHO is not a stable phenotype. Longitudinal data demonstrate that a substantial proportion of metabolically healthy individuals with obesity progress toward metabolic dysfunction over a 5-to-10-year horizon, particularly in the absence of sustained lifestyle modification [119]. This instability has a critical practical implication: biomarker-based phenotyping cannot be a one-time classification exercise. Serial biomarker monitoring—tracking trajectories of adiponectin, inflammatory markers, lipid fractions, and glycemic indices over time—is essential to detect MHO-to-MUO transitions early and trigger proactive intervention before complications become irreversible.

### 7.3. Monitoring Dietary and Lifestyle Interventions: Toward Biomarker-Informed Personalization

One of the most direct and clinically actionable applications of metabolic biomarkers is their use as objective, quantifiable response metrics in dietary and lifestyle intervention trials. Subjective dietary adherence assessments and anthropometric outcomes alone are insufficient to capture the heterogeneous biological responses that characterize real-world interventions. Biomarkers provide the mechanistic granularity required for precision.

The Mediterranean dietary pattern offers a robust illustrative case. In clinical practice, a patient presenting with elevated hs-CRP, reduced adiponectin, increased HOMA-IR, and an unfavorable lipid profile (high TG, low HDL, elevated ApoB) represents a candidate for whom a Mediterranean-pattern intervention can be both prescribed and monitored with biomarker precision [30]. Its adoption is associated with increases in circulating adiponectin; reductions in leptin, TNF-α, IL-6, and hs-CRP; improvements in TG, HDL, LDL, and ApoB profiles; and decreased HOMA-IR and HbA1c—a biomarker signature that aligns mechanistically with its fatty acid composition, fiber content, and antioxidant load [120]. Reassessment at 8–12 weeks provides an objective metabolic response profile that informs whether the intervention is sufficient or requires modification.

Low-carbohydrate dietary approaches similarly produce a characteristic and measurable biomarker trajectory: reductions in fasting TG and insulin resistance indices are typically detectable within two to four weeks, providing early objective confirmation of metabolic engagement [121]. In a patient with predominant hypertriglyceridemia and impaired glucose metabolism, this early biomarker response—or its absence—can guide timely dietary adjustment before anthropometric changes become apparent. Omega-3 fatty acid supplementation produces particularly pronounced and intervention-specific effects on TG and ApoB, making it suitable as an adjunct whose efficacy can be directly tracked through serial lipid panels [24].

The critical clinical advance is to use these biomarker trajectories not merely as outcome measures but as real-time decision tools. Consider a patient adhering to a Mediterranean-pattern diet who fails to show the expected reductions in HOMA-IR or hs-CRP at the 12-week assessment. This non-response pattern defines a clinically distinct subphenotype that warrants mechanistic investigation. Potential drivers include an unfavorable gut microbiota composition with reduced SCFA-producing capacity, genetic variants affecting lipid metabolism or inflammatory signaling, or visceral adipose tissue distribution that sustains chronic low-grade endotoxemia despite dietary improvement. In such cases, biomarker profiling directs the next therapeutic step: microbiota-targeted interventions, intensified physical activity to improve insulin sensitivity independently of diet, or pharmacological adjuncts such as GLP-1 receptor agonists in individuals with persistent hyperinsulinemia [122].

A second illustrative scenario involves post-bariatric surgery patients, in whom dramatic shifts in gut microbiota composition, bile acid profiles, and incretin secretion produce rapid and profound biomarker changes. Serial assessment of miR-122, HOMA-IR, and inflammatory markers in this population allows clinicians to distinguish metabolic responders from non-responders early in the postoperative period, enabling differentiated nutritional rehabilitation protocols before clinical complications emerge.

Biomarker-guided iteration thus transforms a static dietary prescription into a dynamic, responsive therapeutic process—one in which the absence of an expected biological signal is itself a clinically informative finding that drives protocol adaptation [121,122,123,124].

### 7.4. Integration with Omics Technologies: Unlocking the Full Complexity of Metabolic Phenotypes

Traditional biomarker panels, however informative, capture only a fraction of the biological complexity underpinning obesity-related metabolic dysfunction. The integration of multi-omics technologies—metabolomics, proteomics, lipidomics, and metagenomics—represents the next frontier in precision nutrition, with the potential to identify novel biomarkers, elucidate mechanistic pathways, and stratify metabolic phenotypes at a resolution unachievable by conventional clinical assays.

Metabolomics enables the simultaneous quantification of hundreds of small-molecule metabolites in blood, urine, or tissue, providing a real-time snapshot of systemic metabolic flux. Metabolomic signatures—including branched-chain amino acids (BCAAs), acylcarnitines, ceramides, and lysophospholipids—have emerged as early predictors of insulin resistance and T2D risk, independently of conventional clinical markers [118]. BCAA elevations, in particular, reflect impaired skeletal muscle catabolism and mitochondrial dysfunction that precede glycemic deterioration by years, offering a mechanistically grounded early warning signal.

Lipidomics extends beyond the standard lipid panel to characterize the full complexity of the circulating lipidome, including specific ceramide species, lysophospholipids, and triglyceride molecular species. Among these, Ceramide species appear particularly informative. Elevated circulating ceramides are associated with hepatic lipid accumulation, endothelial dysfunction, and increased cardiovascular risk and may outperform conventional lipid markers in predicting adverse cardiometabolic outcomes [125]. Alterations in lysophospholipids, including Lysophosphatidylcholine, have similarly been linked to inflammation, insulin resistance, and adipose tissue remodeling, suggesting their potential value as markers of metabolic phenotype.

Protein biomarkers derived from adipose tissue and systemic inflammatory signaling represent another emerging category. Adipokines such as Adiponectin and Leptin remain central regulators of energy homeostasis and insulin sensitivity, but additional adipose-secreted proteins are increasingly being recognized [116,126]. Candidates, including Retinol-binding protein 4 and Fibroblast Growth Factor 21, have shown associations with hepatic steatosis, metabolic stress, and impaired glucose metabolism, suggesting a broader panel of adipose-derived signals that may better capture metabolic heterogeneity among individuals with obesity [127].

Gut metagenomics adds a further dimension by characterizing the microbial determinants of metabolic response. Inter-individual variation in dietary response is substantially mediated by gut microbiota composition, which governs the production of short-chain fatty acids, secondary bile acids, TMAO, and other metabolites that directly modulate adipose tissue function, insulin sensitivity, and lipid metabolism [92]. Conversely, metabolites such as Trimethylamine *N*-oxide have been associated with increased cardiometabolic risk and altered lipid metabolism. Variability in the production of these microbial metabolites may therefore help explain inter-individual differences in metabolic response to diet [102]. Taken together, these emerging biomarkers highlight the multifactorial nature of obesity-related metabolic dysfunction, encompassing disturbances in amino acid metabolism, lipid signaling pathways, adipose tissue endocrinology, and host–microbiome interactions. Rather than replacing established clinical markers, these candidate biomarkers may extend current diagnostic frameworks by enabling earlier detection of metabolic impairment, refining cardiometabolic risk stratification, and identifying biologically distinct subphenotypes within obesity that may benefit from targeted nutritional or therapeutic interventions.

### 7.5. Barriers to Clinical Implementation

The gap between biomarker science and clinical practice is not a knowledge gap—it is a structural one. Several interconnected barriers impede implementation, and acknowledging their nature more precisely is essential to addressing them effectively.

Analytical heterogeneity remains the most pervasive obstacle. Adipokines such as adiponectin and leptin yield systematically discrepant values across immunoassay platforms due to differences in antibody epitope recognition, matrix effects, and calibration standards [128]. Inflammatory cytokines at sub-picomolar concentrations are acutely sensitive to pre-analytical variables—venipuncture time, tube type, centrifugation protocols, and freeze–thaw cycles—that are rarely standardized across clinical sites [129]. Without harmonized measurement protocols and certified reference materials, biomarker data are effectively non-comparable across institutions, rendering multicenter validation impossible. The contrast with lipid measurement is instructive: the adoption of standardized LDL-C reporting, anchored by the CDC Lipid Standardization Program and enforced through the National Cholesterol Education Program (NCEP) guidelines, transformed a similarly heterogeneous landscape into a reliable clinical tool over two decades [130]. No equivalent standardization infrastructure yet exists for adipokine or cytokine panels.

Population-specific reference ranges remain critically underdeveloped. Biomarker concentrations vary substantially by age, sex, ethnicity, menopausal status, and adipose tissue distribution [131], yet most published reference intervals derive from predominantly White, European populations. Universal cutoff values applied to diverse populations introduce systematic misclassification that undermines both diagnosis and risk stratification. This limitation is not merely theoretical: the 2023 American Diabetes Association Standards of Care explicitly acknowledge that HbA1c thresholds perform differently across racial and ethnic groups and recommend supplemental fasting glucose or continuous glucose monitoring in populations where hemoglobin variants distort glycated hemoglobin assays [132]. A comparable recognition of population-specific variability has not yet been incorporated into obesity-related biomarker guidance by major bodies such as the European Association for the Study of Obesity (EASO) or the Endocrine Society.

Cost and infrastructure constraints restrict comprehensive biomarker profiling to academic and research settings. Advanced panels—specific adipokines, cytokine multiplex assays, ApoB quantification, and lipoprotein particle sizing—are not reimbursed by most national healthcare systems and require laboratory expertise unavailable in primary care or community health settings. The trajectory of HbA1c offers a relevant precedent here as well: once an exclusively research-grade assay, it was progressively standardized, point-of-care formats were developed, and reimbursement followed guideline endorsement—a process that took roughly thirty years but ultimately enabled near-universal clinical access. Achieving equitable implementation of cardiometabolic biomarker panels will require analogous investment in point-of-care assay development, dried blood spot methodologies, and cost-reduction strategies, accompanied by deliberate inclusion of low-resource settings in implementation research.

**Figure 1:**
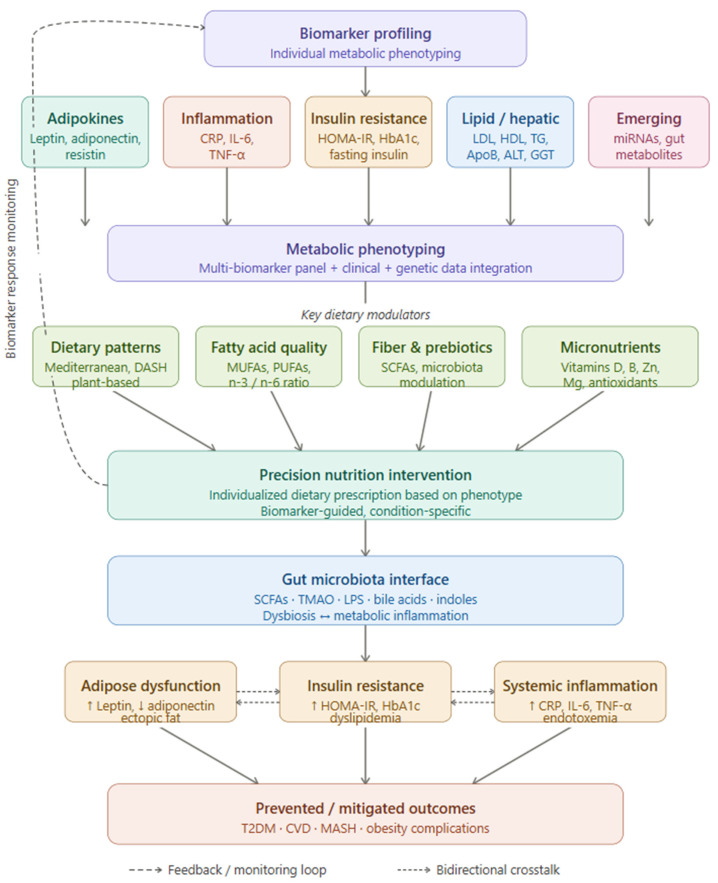
Biomarker-based precision nutrition framework for obesity management.

Finally, and perhaps most fundamentally, the field lacks high-quality randomized evidence that biomarker-guided treatment protocols improve hard clinical outcomes—not merely surrogate biomarker values—compared to standard care. The experience of B-type natriuretic peptide (BNP)-guided therapy in heart failure is instructive in this respect: despite strong biological plausibility and widespread adoption of BNP as a prognostic marker, subsequent randomized trials of BNP-guided treatment intensification produced inconsistent results on mortality, prompting guidelines to moderate earlier enthusiasm and restrict guidance-directed use to specific patient subgroups [133]. Obesity medicine risks repeating this pattern if biomarker-guided algorithms are implemented before outcome evidence matures. Pragmatic trials embedding biomarker-guided personalization within real-world obesity management pathways—modeled on adaptive platform designs such as those used in the RECOVERY or REMAP-CAP trials—with pre-specified outcome hierarchies and health-economic analyses are urgently needed to build the evidence base required for guideline adoption and reimbursement policy (Figure 1).

## 8. Future Directions and Research Priorities

International harmonization of measurement methods and quality control procedures would enhance comparability across studies and facilitate broader clinical application. Longitudinal studies are essential to clarify how biomarkers change over time, predict metabolic and cardiovascular outcomes, and respond to dietary and lifestyle interventions. Prospective research with repeated measurements can identify transition points between metabolically healthy and unhealthy obesity and evaluate intervention effectiveness. Advanced analytical tools, including trajectory modeling and machine learning, may further refine predictive capacity. Importantly, such studies must include diverse populations to ensure generalizability and equity. Ultimately, the goal of biomarker research is to support personalized obesity management. Integrating biomarker data with genetic, epigenetic, microbiome, metabolomic, and behavioral information—along with emerging technologies such as continuous glucose monitoring and wearable devices—can enable more precise, individualized interventions. However, successful implementation will require healthcare infrastructure development, multidisciplinary training, and equitable access to diagnostic tools.

In conclusion, nutritional and metabolic biomarkers offer a powerful framework for understanding the complexity of obesity and advancing precision nutrition. Continued research, standardization, validation, and clinical integration are crucial to improving metabolic health outcomes in individuals living with obesity.

## 9. Conclusions

Nutritional and metabolic biomarkers provide a powerful and physiologically grounded framework for characterizing the heterogeneous metabolic landscape of obesity. The biomarker categories reviewed here—adipokines, inflammatory cytokines, insulin resistance indices, lipid fractions, hepatic enzymes, microRNAs, gut microbiota-derived metabolites, and micronutrient markers—collectively capture distinct but interrelated axes of metabolic dysfunction. Their integration into multi-biomarker panels enables meaningful risk stratification, early identification of metabolic deterioration, and monitoring of dietary and lifestyle intervention responses. Continued investment in standardization, longitudinal validation across diverse populations, and the development of clinically implementable precision nutrition tools will be essential to realizing the full translational potential of biomarker-guided obesity management.

## Figures and Tables

**Table 1:** Inflammatory and Adipose Tissue-Derived Biomarkers.

Biomarker	Primary Source	Main Biological Function	Clinical Relevance
Leptin	Mature adipocytes (white adipose tissue)	Regulates appetite and energy expenditure via hypothalamic signaling; reflects fat mass	Elevated in obesity; leptin resistance associated with weight gain, insulin resistance, and cardiovascular risk
Adiponectin	Mature adipocytes (especially subcutaneous fat)	Enhances insulin sensitivity; stimulates fatty acid oxidation (AMPK, PPARα); anti-inflammatory and anti-atherogenic	Reduced in obesity, type 2 diabetes, MASLD, and cardiovascular disease; protective metabolic biomarker
Resistin	Adipocytes and immune cells (mainly macrophages in humans)	Promotes inflammation and impairs insulin signaling; stimulates hepatic LDL receptor degradation and ApoB-containing lipoprotein secretion	Increased in obesity and metabolic syndrome; linked to insulin resistance, systemic inflammation, and atherogenic dyslipidemia
TNF-α	Adipose tissue macrophages; hypertrophic adipocytes	Inhibits insulin signaling; promotes lipolysis and inflammation; suppresses LPL-mediated TG clearance and stimulates hepatic VLDL-ApoB secretion	Elevated in obesity; contributes to insulin resistance, raised TG, reduced HDL, and chronic low-grade inflammation
IL-6	Adipocytes and stromal-vascular immune cells	Regulates immune responses; stimulates hepatic acute-phase protein synthesis; promotes hepatic VLDL production and ApoB secretion	Elevated in obesity and insulin resistance; induces CRP production, drives atherogenic dyslipidemia, and predicts cardiometabolic risk
IL-1β	Activated macrophages within adipose tissue	Potent pro-inflammatory cytokine; disrupts insulin signaling and β-cell function	Associated with metabolic inflammation, insulin resistance, and progression to type 2 diabetes
*C*-reactive protein (CRP)	Liver (induced by IL-6 from adipose tissue)	Acute-phase inflammatory marker	Widely used clinical biomarker of obesity-associated inflammation and cardiovascular disease risk
HDL cholesterol	Liver and intestine; matured in circulation via reverse cholesterol transport	Mediates reverse cholesterol transport from peripheral tissues to the liver; exerts anti-inflammatory and antioxidant effects on the vasculature; supports adiponectin-driven lipid utilization	Reduced in obesity, insulin resistance, and metabolic syndrome; low HDL is an independent predictor of cardiovascular disease risk and reflects adipose tissue dysfunction
LDL cholesterol	Derived from VLDL catabolism in circulation; regulated by hepatic LDL receptor activity	Transports cholesterol to peripheral tissues; excess LDL, particularly small dense particles, promotes endothelial dysfunction and atherosclerotic plaque formation	Elevated in dyslipidemia associated with adipose tissue inflammation and Western dietary patterns; a primary therapeutic target for cardiovascular risk reduction
Triglycerides (TG)	Liver (VLDL-TG) and intestine (chylomicrons); clearance mediated by LPL in adipose and muscle tissue	Primary form of circulating fatty acid transport and energy storage; elevated TG reflects excess hepatic VLDL secretion driven by adipose-derived free fatty acid flux and DNL	Elevated in obesity, insulin resistance, and metabolic syndrome; hypertriglyceridemia reflects impaired adipose LPL activity and is associated with increased cardiovascular risk, particularly when accompanied by low HDL
Apolipoprotein B (ApoB)	Liver (ApoB-100 on VLDL, IDL, LDL) and intestine (ApoB-48 on chylomicrons)	Structural and functional protein of all atherogenic lipoprotein particles; one ApoB molecule per particle makes it a direct measure of total circulating atherogenic particle number	Elevated in obesity and adipose tissue dysfunction due to increased hepatic VLDL-ApoB secretion driven by excess free fatty acid flux, inflammation, and reduced adiponectin; superior to LDL cholesterol alone in predicting residual cardiovascular risk

**Table 2:** Summary of clinically relevant circulating miRNA biomarkers in obesity.

miRNA	Metabolic Association	Nutritional Modulation
miR-122	Hepatic lipid metabolism; elevated in MASLD/MASH and T2D; correlates with ALT	Reduced by omega-3 fatty acids and Mediterranean diet
miR-126	Vascular integrity; reduced levels linked to T2D and CVD risk	Increased by physical activity and *n*-3 PUFA supplementation
miR-375	Pancreatic β-cell function; elevated in obesity; impaired insulin secretion	Modulated by caloric restriction and low-GI diets
miR-222	Adipogenesis regulator; elevated in obesity; associated with insulin resistance	Influenced by dietary fat quality (SFA vs. MUFA/PUFA)
miR-29a	Glucose metabolism; dysregulated in T2D and liver fibrosis	Reduced by polyphenol-rich diets
miR-144	Insulin sensitivity; reduced expression linked to impaired glucose homeostasis	Upregulated by *n*-3 PUFAs and dietary fiber
miR-103	Insulin sensitivity; elevated levels impair INSR signaling in the liver and adipose	Suppressed by caloric restriction

**Table 3:** Summary of key gut microbiota-derived biomarkers in obesity.

Biomarker	Metabolic Association	Dietary Modulation
SCFAs (butyrate, propionate, acetate)	Intestinal barrier integrity; insulin sensitivity; anti-inflammatory; appetite regulation	Increased by dietary fiber, resistant starch, legumes, whole grains
TMAO	CVD risk (contested); insulin resistance; systemic inflammation	Reduced by plant-based/Mediterranean diet; elevated by red meat
LPS (endotoxemia marker)	Systemic inflammation; insulin resistance; hepatic steatosis	Reduced by fiber + *n*-3 PUFAs; worsened by high-fat Western diet
Secondary bile acids	FXR/TGR5 signaling; lipid absorption; glucose metabolism	Modulated by dietary fiber and fat quality
Indole metabolites	Gut barrier integrity; mucosal immunity; AhR signaling	Enhanced by diverse plant-food intake and probiotics

**Table 4:** Key micronutrient biomarkers in obesity: clinical relevance and dietary modulation.

Micronutrient	Biomarker Measured	Deficiency Consequences in Obesity	Dietary Sources
Vitamin D	25(OH)D serum	Impaired insulin secretion; elevated PTH; increased inflammatory cytokines; reduced adiponectin	Oily fish, fortified dairy, sunlight
Iron	Serum ferritin; transferrin saturation; sTfR	Chronic fatigue; impaired mitochondrial function; hepcidin dysregulation	Red meat, legumes, fortified cereals + vitamin C
Vitamin B12	Serum B12; holotranscobalamin; MMA	Hyperhomocysteinemia; neurological dysfunction; impaired one-carbon metabolism	Meat, fish, dairy, eggs; supplement in vegans/metformin users
Folate	Serum/RBC folate; homocysteine	Impaired DNA methylation; elevated homocysteine; CVD risk	Leafy greens, legumes, fortified grains
Magnesium	Serum Mg; erythrocyte Mg	Impaired insulin signaling; increased inflammatory markers; T2D and MetS risk	Nuts, seeds, whole grains, leafy greens
Zinc	Serum zinc; alkaline phosphatase	Impaired immune function; altered adipokine secretion; oxidative stress	Meat, shellfish, legumes, nuts

## Data Availability

No new data were created or analyzed in this study. Data sharing is not applicable to this article.

## Abbreviations

The following abbreviations are used in this manuscript:

ALT	Alanine aminotransferase
AMPK	AMP-activated protein kinase
AMP	Adenosine monophosphate
ApoB	Apolipoprotein B
AST	Aspartate aminotransferase
ATGL	Adipose triglyceride lipase
BMI	Body mass index
ChREBP	Carbohydrate response element-binding protein
CRP	*C*-reactive protein
CVD	Cardiovascular disease
CYP7A1	Cytochrome P450 family 7 subfamily A member 1
DGAT	Diacylglycerol acyltransferase
DHA	Docosahexaenoic acid
DNL	De novo lipogenesis
EPA	Eicosapentaenoic acid
FODMAP	Fermentable oligosaccharides, disaccharides, monosaccharides and polyols
FXR	Farnesoid X receptor
GLUT4	Glucose transporter type 4
HbA1c	Glycated hemoglobin (hemoglobin A1c)
HDL	High-density lipoprotein
HDL-C	High-density lipoprotein cholesterol
HOMA-IR	Homeostatic model assessment of insulin resistance
HPA	Hypothalamic–pituitary–adrenal axis
hs-CRP	High-sensitivity *C*-reactive protein
HSL	Hormone-sensitive lipase
IL-1β	Interleukin-1 beta
IL-6	Interleukin-6
LDL	Low-density lipoprotein
LDL-C	Low-density lipoprotein cholesterol
LPL	Lipoprotein lipase
LPS	Lipopolysaccharide
MAFLD	Metabolic dysfunction-associated fatty liver disease
MASH	Metabolic dysfunction-associated steatohepatitis
MASLD	Metabolic dysfunction-associated steatotic liver disease
MGL	Monoacylglycerol lipase
MHO	Metabolically healthy obesity
MUFAs	Monounsaturated fatty acids
MUO	Metabolically unhealthy obesity
NF-κB	Nuclear factor kappa-light-chain-enhancer of activated B cells
NHANES	National Health and Nutrition Examination Survey
PKA	Protein kinase A
PPARα	Peroxisome proliferator-activated receptor alpha
PUFA	Polyunsaturated fatty acid
PUFAs	Polyunsaturated fatty acids
SCFA	Short-chain fatty acid
SCFAs	Short-chain fatty acids
SREBP1	Sterol regulatory element-binding protein 1
SREBP2	Sterol regulatory element-binding protein 2
SVF	Stromal-vascular fraction
T2D	Type 2 diabetes
T2DM	Type 2 diabetes mellitus
TAG	Triacylglycerol
TGR5	Takeda G protein-coupled receptor 5
TMAO	Trimethylamine *N*-oxide
TNF-α	Tumor necrosis factor alpha
TRL	Triglyceride-rich lipoprotein
UPF	Ultra-processed food
VLDL	Very-low-density lipoprotein
